# The Diabetes Attitudes Wishes and Needs (DAWN)‐SMI study: A cross sectional comparison of the psychosocial impact of diabetes in adults with and without severe mental illness

**DOI:** 10.1111/dme.70126

**Published:** 2025-08-16

**Authors:** Richard I. G. Holt, Heather Peyrot‐Stuckey, Dankmar Böhning, Jo Taylor, Najma Siddiqi

**Affiliations:** ^1^ Human Development and Health, Faculty of Medicine University of Southampton Southampton UK; ^2^ College of Medicine Pennsylvania State University Hershey Pennsylvania USA; ^3^ Mathematical Sciences and Southampton Statistical Sciences Research Institute University of Southampton Southampton UK; ^4^ Department of Health Sciences University of York York UK; ^5^ Hull York Medical School York UK; ^6^ Bradford District Care NHS Foundation Trust Bradford UK

**Keywords:** mental health, psychosocial

## Abstract

**Aims:**

People with severe mental illness (SMI) are 2–3 times more likely to have diabetes than the general population. Little is known about the impact of living with diabetes for people with SMI. This study investigates psychosocial problems and diabetes self management for people with SMI and diabetes.

**Methods:**

We compared cross sectional survey data collected from 258 adults with diabetes and SMI in England with 500 adults with diabetes from the UK sample of the second Diabetes Attitudes, Wishes and Needs study (DAWN2). Effect size (ES) tests were used to quantify differences between the two samples adjusted for diabetes type, age, gender, treatment, treatment duration, diabetes complications and co‐morbidities to achieve comparability of the two samples.

**Results:**

Compared to the DAWN2‐UK sample, people with diabetes and SMI reported poorer quality of life (WHOQOL ES −0.3 (CI −0.5, −0.1), *p* < 0.001), mental well‐being (ES −13.4 (CI −17.3, −9.5), *p* < 0.001) and increased diabetes distress (PAID5 ES 1.6 (CI 0.9,2.3), *p* < 0.001). While people with diabetes and SMI reported a negative impact from diabetes, their SMI had a greater impact on their lives than diabetes (mental illness impact profile 2.6 ± 1.1 vs. diabetes impact profile 3.4 ± 1.0, *p* < 0.001). People with SMI reported being less engaged in self management than the DAWN2‐UK population (SDSCA‐6; ES −0.4 (CI −0.7, −0.1), *p* = 0.01).

**Conclusions:**

The psychosocial impact of diabetes is greater for people with SMI. To reduce inequalities in diabetes outcomes, people with SMI and diabetes require tailored support for diabetes management that considers the additional challenges associated with living with a severe mental illness.

## Graphical abstract

People with severe mental illness (SMI) and diabetes face greater psychosocial challenges, higher diabetes distress, and poorer self‐management than those with diabetes alone, highlighting the need for tailored, person‐centred diabetes care that addresses the unique challenges of living with diabetes and SMI to reduce health inequalities and improve outcomes.

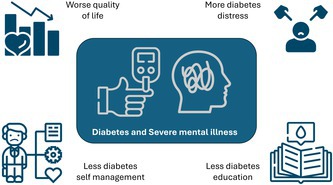

**What's new?**

**What is already known?**

- People with severe mental illness (SMI) die on average 15–20 years younger than people without SMI, are 2–3 times more likely to develop diabetes and are at higher risk of suboptimal diabetes outcomes.

**What has this study found?**

- People with SMI and diabetes experience higher psychosocial burden, higher levels of diabetes distress and receive less support, less person‐centred care and less diabetes education compared to people with diabetes without SMI.

**What are the implications of this study?**

- People with SMI and diabetes need to have access to adequate, person‐centred support that addresses the issues associated with living with SMI and diabetes.

## 1 BACKGROUND

Individuals with severe mental illness (SMI), including schizophrenia and bipolar disorder, experience a markedly reduced life expectancy, estimated at 15–20 years shorter than the general population.^1^, ^2^ A substantial portion of this mortality gap is attributed to the higher prevalence of physical health co‐morbidities, particularly type 2 diabetes. An umbrella review of 245 observational studies reported an elevated prevalence of type 2 diabetes in many psychiatric populations, with rates of 10%–11% among people with psychosis, bipolar illness and schizophrenia.^3^ Another comprehensive synthesis of systematic reviews confirmed that use of antipsychotics was associated with a 94% increased risk for developing type 2 diabetes, independent of demographic and lifestyle factors.^4^

Multiple factors contribute to the elevated diabetes risk among people with SMI. These include a greater likelihood of having a family history of diabetes,^5^ the metabolic side effects of antipsychotics, physical inactivity, poor diet, high smoking rates^6^ and persistent socioeconomic inequalities.^7^ Furthermore, individuals with SMI often experience worse clinical outcomes and higher rates of diabetes‐related complications.^8^ For example, a nationwide cohort study in Denmark found that people with SMI and type 2 diabetes had an increased risk of developing nephropathy (15%), cardiovascular disease (10%–18%) and amputation (15%) compared to individuals with diabetes alone. The disparities were most pronounced in younger adults.^9^ This can partly be attributed to systemic healthcare issues, which include inadequate physical health monitoring, ‘diagnostic overshadowing’, where physical symptoms are misattributed to mental illness, and fragmentation of care due to unclear inter‐service responsibilities.^10^

In addition to these barriers, people with SMI often face cognitive, emotional and social challenges that hinder effective health self management.^11^, ^12^ Consequently, comorbid diabetes may impose a particularly high burden on this group. Although various risk factors for poor physical health outcomes in SMI are well documented, there remains limited understanding of the *psychosocial* impact of living with both SMI and diabetes. Both conditions are independently associated with substantial disease burden and reduced health‐related quality of life (HRQoL),^13^, ^14^ yet the specific lived experience of managing these dual diagnoses has not been adequately explored.

Understanding the psychosocial dimensions of comorbid SMI and diabetes is critical to improving care and tailoring interventions for this vulnerable population. However, to date, no published studies have directly examined this issue.

## 2 STUDY AIM AND OBJECTIVES

This study aimed to explore the psychosocial impact of living with both diabetes and SMI. Specifically, we assessed: (1) health‐related quality of life (HRQoL), (2) mental well‐being, (3) diabetes‐related distress and (4) the impact of both conditions across various life domains. We also evaluated diabetes self management practices, sources of diabetes‐related support and participation in structured diabetes education. Finally, we compared our findings with those from individuals living with diabetes alone, using data from the UK participants of the second Diabetes Attitudes, Wishes and Needs (DAWN2) study.^13^

## 3 METHODS

### 3.1 Study design

We conducted a cross sectional survey of adults in England with comorbid SMI and diabetes, using an adapted version of the DAWN2 questionnaire.^15^ Findings from this survey were compared with data from the original DAWN2‐UK study, which included 500 participants living with diabetes.^13^

### 3.2 The DAWN‐SMI Survey

To enhance the feasibility and accessibility for individuals with SMI, we developed a shortened version of the UK DAWN2 questionnaire.^15^ While certain sections were removed to reduce respondent burden, the content of individual questions and validated measures was not modified, ensuring comparability with the original DAWN2‐UK dataset. Adaptation decisions were informed through consultation with individuals with lived experience of both SMI and diabetes, as well as members of the original DAWN2 research team, who advised on the relevance and utility of specific survey components. Additional items were included to capture information specific to the SMI population, including SMI diagnosis, treatment and perceived illness burden. To assess the impact of mental illness, the DAWN Impact of Diabetes Profile question was changed from ‘How does *diabetes* currently impact the following aspects of your life’ to ‘How does *mental illness* currently impact the following aspects of your life’. The revised question retained the same six life aspects and response options, ranging from very negative to very positive, including a non‐applicable option. Open‐ended (free‐text) questions were also added to explore personal experiences of managing diabetes alongside SMI. The measures included in the survey are summarised in Table 1.

**TABLE 1:** Survey measures used in the DAWN‐SMI survey.

Domain	Measure(s)	Description of measure
Health‐related quality of life (HRQoL)	EQ‐Visual Analogue Scale (EQ‐5D VAS)^24^	A vertical scale with best and worst imaginable health state endpoints to allow participants to rate their own health, and was the main outcome in DAWN2
Mental well‐being	World health Organisation (WHO) Well‐being index (WHO‐5)^25^	5‐item scale used widely in research and clinical practice as a valid and reliable measure of depressive symptoms that allows for severity to be assessed
Diabetes distress	Problem Area in Diabetes Questionnaire (PAID‐5)^26^	5‐item scale which has been validated as a reliable measure of diabetes distress with greater validity than the original 20‐item scale
Congruence of care with the Chronic Care Model	Patient Assessment of Chronic Illness Care^27^	A 20‐item scale that measures used to assess care against the Chronic Care Model. The scale is based around five subscales: (a) patient activation, (b) delivery system design and decision support, (c) goal setting and tailoring, (d) problem‐solving and contextual counselling, (e) follow‐up and coordination
Impact of diabetes/SMI	DAWN Impact of Diabetes Profile (DIDP)/DAWN Impact of Mental Illness Profile (DIMP)^15^	Asks people with diabetes and SMI to rate how negatively or positively diabetes and SMI impacts on different aspects of life
Diabetes empowerment	Diabetes Empowerment Scale‐DAWN Short Form (DES‐DSF)^15^	Inspired by items from the original Diabetes Empowerment Scale (DES‐SF) and other work on diabetes empowerment
Self management of diabetes	Summary of Diabetes Self Care Activities (SDSCA‐6)^28^	Captures how well participants manage different aspects of their diabetes over a 7‐day period
Sources of support	DAWN Support for Diabetes Self Management Profile^15^	Explores perceived level of support for self management
Diabetes structured education provision	Original items from DAWN2 survey^15^	Explores use of diabetes education resources

Participants were offered multiple options for completing the questionnaire—by post, online, in person with a researcher or via telephone—to ensure accessibility and to accommodate those who required support or limited digital literacy. The online version of the survey was administered using Qualtrics, which was also used to enter responses collected during telephone interviews.

### 3.3 DAWN2‐UK data

To compare findings from people with SMI and diabetes to those with diabetes alone, we used data from the 500 UK participants in the DAWN2‐UK study.^13^ This dataset included demographic and screening information suitable for comparative analysis. While DAWN2‐UK did not explicitly exclude individuals with SMI, it also did not collect data on comorbid mental health diagnoses. However, given that recruitment for DAWN2‐UK was conducted via internet and telephone, it is unlikely that individuals with SMI were well represented in that sample.^15^ Full details of the DAWN2‐UK recruitment methodology have been previously published.^15^

The DAWN2‐UK sample comprised 81 adults with type 1 diabetes and 419 with type 2 diabetes. Exclusion criteria included individuals under 18 years of age, those diagnosed with diabetes within the past year and pregnancy‐related diabetes.

### 3.4 DAWN‐SMI Survey

For the DAWN‐SMI survey, eligible participants were adults (aged ≥18 years old) diagnosed with both SMI and diabetes, with both conditions present for at least 1 year. We included individuals with schizophrenia, schizoaffective disorder, bipolar disorder or other non‐organic psychotic disorders, alongside a diagnosis of either type 1 diabetes or type 2 diabetes. Exclusion criteria included current inpatient treatment, gestational diabetes and lack of capacity to provide informed consent.

Given the absence of a national registry of individuals with both SMI and diabetes, we employed purposive sampling strategies to maximise sample diversity and representativeness. Participants were recruited from various geographical regions across England, including both urban and rural areas, to reflect a range of demographic profiles and local health and social care commissioning arrangements. Recruitment sites included both NHS mental health trusts and general practices, ensuring inclusion of individuals receiving care from either primary care providers or specialist mental health services.

In general practices, eligible participants were identified through electronic health record searches. Within mental health trusts, care coordinators and psychiatrists identified eligible individuals from their caseloads. Informed consent was obtained from all participants prior to their involvement in the study.

## 4 STATISTICAL ANALYSIS

The planned comparison of the DAWN2‐UK and DAWN‐SMI cohorts focused on three areas that were addressed in separate analyses: (1) differences in health‐related quality of life (HRQoL: EQ‐5D Visual Analogue Scale), (2) mental well‐being (WHO‐5, PAID‐5) and (3) diabetes‐related distress and experiences of diabetes self management (DES‐DSF; SDSCA‐6; DIDP).

Descriptive statistics were used to summarise findings for all variables. Because of the sampling method used in the DAWN2‐UK study, the DAWN‐SMI cohort had a much higher prevalence of type 2 diabetes.^15^ To achieve better comparability, we evaluated the between‐sample differences controlling for a broad set of potentially confounding factors, including type of diabetes, duration of diabetes, gender, age, treatment and diabetes‐related complications and co‐morbidities. We used effect size estimates from linear regression, logistic regression or Poisson regression to quantify the differences between the DAWN‐SMI and the DAWN2‐UK samples.

For outcomes measured on a continuous or ordinal scale, we used linear regression modelling to assess between‐group differences for two reasons. First, it includes the effect variable of interest (DAWN2‐UK vs. DAWN‐SMI), and second, it allows us to include several potential confounding variables to achieve valid comparisons between the two cohorts.

The model was fitted for each outcome of interest (denoted Y) as follows:

$$\begin{array}{cc} Y\; & =\alpha +\beta_{1}\;\text{effect of interest}+\gamma_{1}\;{\text{confounder}}_{1}+\ldots .\\ & +\gamma_{1}\;{\text{confounder}}_{k}+\text{error}, \end{array}$$

where:

- Effect of interest indicates cohort membership (DAWN2‐UK vs. DAWN‐SMI),
- Age, gender, type and duration of diabetes, diabetes‐related complications and co‐morbidities are covariates identified a priori as potential confounders,
- *α* is the intercept,
- *β*_1_ quantifies the adjusted effect of group membership,
- *γ*_1_ through *γ*_k_ represent effects of individual confounders.

To ensure consistency across models, the same set of confounding variables was included in all regression analyses. This strategy enabled comparisons between DAWN‐UK and DAWN‐SMI cohorts at equivalent levels of the covariates, thereby isolating the effect of group membership. Statistical significance of the group variable (β1) was used to evaluate cohort differences in the outcome Y.

For binary response variables such as *smoking*, logistic regression was used, whereas for count variables such as *the number of people talked to about diabetes*, Poisson regression modelling was employed. Again, we adjusted for the same set of covariates as for linear regression modelling.

All analyses were conducted using Stata 18.5.

## 5 RESULTS

### 5.1 DAWN‐SMI participant characteristics and diabetes profile

263 individuals with coexisting diabetes and SMI responded to the survey. Of these, 195 were recruited through NHS mental health trusts and 68 through general practice. Data from four respondents were excluded due to missing or unusable information, resulting in a final analytical sample of 257 participants.

The mean age of participants was 55.4 years (range: 22–87). The mean age at diagnosis for diabetes was 43.2 years, and for SMI, 30.4 years. 57% were men, and 98% had a diagnosis of type 2 diabetes. The commonest mental health diagnosis was schizophrenia or schizoaffective disorder, reported by 60% of participants.

In terms of diabetes management, two‐thirds of respondents reported using oral antidiabetes agents, while 21% were using insulin. Participants also reported high levels of comorbid psychological and sleep‐related difficulties: 79% had experienced depression, and 65% reported problems with sleep. 87% of those with depression indicated that it had begun prior to their diabetes diagnosis.

A summary of participant characteristics from the DAWN‐SMI survey and comparison with the original DAWN2‐UK population is presented in Table 2.

**TABLE 2:** Characteristics of people with diabetes with and without severe mental illness.

People with diabetes	DAWN‐SMI cohort	UK DAWN2 cohort
	Diabetes and severe mental illness	Diabetes but no severe mental illness
	*n* (%) or mean (SD, *n*)	*n* (%) or mean (SD, *n*)
Age (years)	55.4 (10.7, 257)	57.9 (13.1, 500)
Sex *n*, %		
Men	147 (57%)	284 (57%)
Women	109 (43%)	216 (43%)
BMI (kg/m^2^)	33.8 (7.1, 244)	30.7 (6.7, 489)
Current smokers (*n*, %)	106 (42%)	92 (18%)
Type of severe mental illness		
Schizophrenia/schizoaffective disorder	153 (60%)	
Bipolar disorder	79 (31%)	
Depression	87 (34%)	
Other	43 (17%)	
Not sure	2 (1%)	
Duration of severe mental illness (years)	25.0 (13.5, 251)	
Mental health treatment, *n*, %		
Talking therapies (e.g. counselling)	61 (24%)	
Oral therapies	243 (94%)	
Injectable therapies	26 (10%)	
Diet and Exercise	91 (35%)	
Alternative medicine	10 (4%)	
Other	17 (7%)	
None	4 (2%)	
Type of diabetes *n*, %		
Type 1 diabetes	6 (2%)	81 (16%)
Type 2 diabetes	252 (98%)	419 (84%)
Diabetes duration (years)	12.2 (11.2, 253)	13.5 (12.8, 500)
Glucose lowering treatment, *n*, %		
Diet and exercise	165 (64%)	273 (55%)
Alternative medicine	5 (2%)	22 (4%)
Oral antidiabetes agents	207 (62%)	260 (52%)
Insulin	55 (21%)	231 (46%)
Other injectable diabetes medications	4 (2%)	15 (3%)
Complications/co‐morbidities, *n*, %		
No complications/co‐morbidities	20 (8%)	120 (24%)
With complications/co‐morbidities		
Stroke	21 (8%)	29 (6%)
Foot ulcers	27 (11%)	42 (8%)
Lower extremity amputation	2 (1%)	6 (1%)
Kidney disease	31 (12%)	54 (11%)
Eye damage	69 (27%)	124 (25%)
Nerve damage	44 (17%)	89 (18%)
Sexual dysfunction	64 (25%)	133 (27%)
Heart disease	30 (15%)	82 (16%)
Depression	204 (79%)	157 (31%)
Sleeping problems	167 (65%)	205 (41%)

### 5.2 Psychosocial measures

**FIGURE 1:**
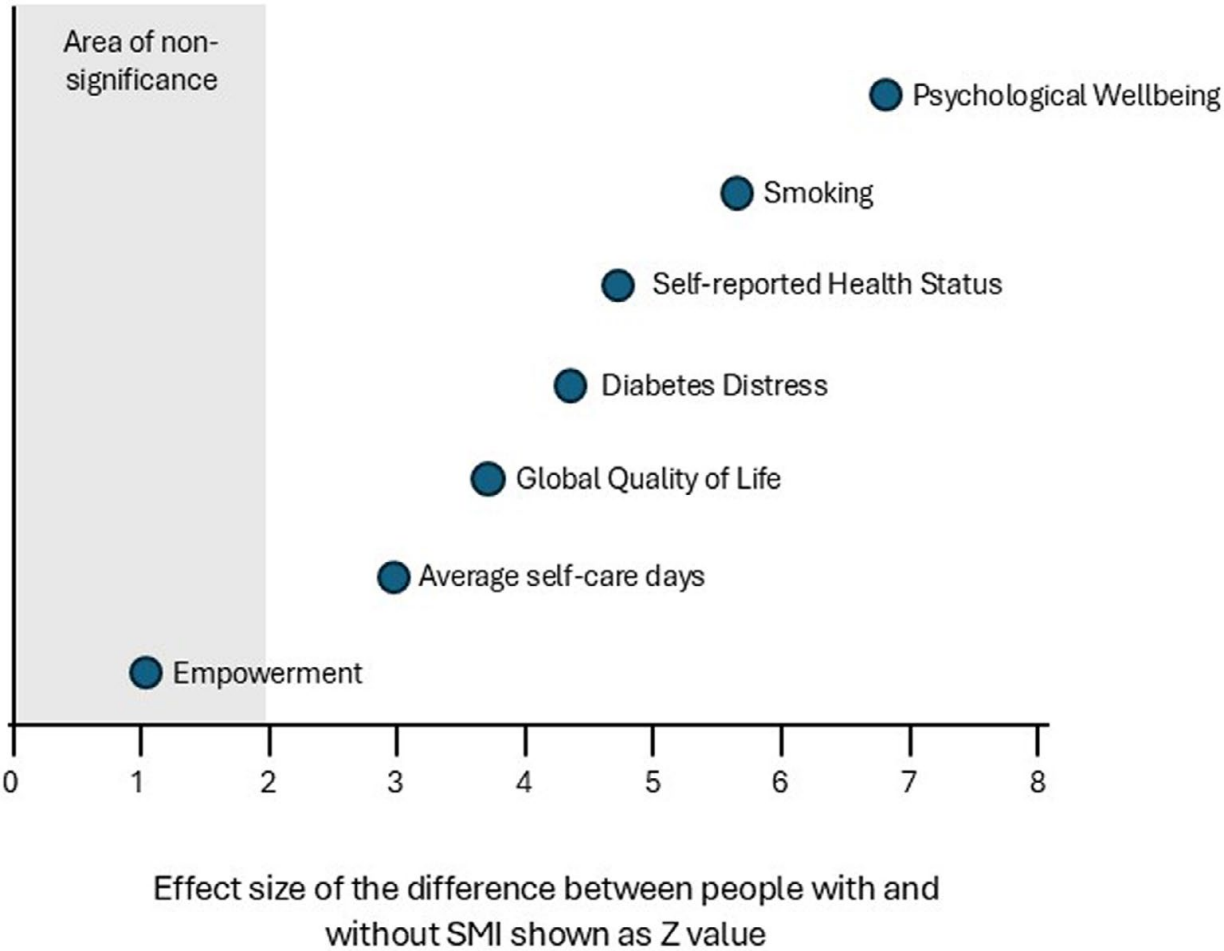
Graphical representation of the effect size of the difference between people with diabetes and SMI (DAWN‐SMI cohort) and diabetes alone (DAWN‐UK), shown as a Z‐value. In the original scales, higher scores for diabetes distress and smoking indicate worse health status, while lower scores for all other measures indicate worse health status. We have aligned the scaling for all measures so that worse health outcome is shown in the same direction.

Table 3 presents a summary of the psychosocial measures among individuals with diabetes and SMI, compared with respondents from the DAWN2‐UK study. Individuals with diabetes and SMI reported lower health‐related quality of life and psychological well‐being, as well as higher levels of diabetes‐related distress. Diabetes had a greater impact on physical health, work and emotional well‐being in people with diabetes and SMI than those with diabetes alone. However, the SMI had a greater impact than diabetes on the lives of those with diabetes and SMI (DAWN Impact of mental illness profile (DIMP) 2.6 ± 1.1 vs. DAWN Impact of diabetes profile (DIDP) 3.4 ± 1.0, *p* < 0.001). The magnitude of the difference between people with and without SMI is shown as a z‐value in Figure 1.

**TABLE 3:** Key psychosocial and healthcare indicators for people with diabetes with and without severe mental illness.

	DAWN‐SMI cohort	DAWN‐2 UK cohort	
Psychosocial measure	Diabetes and severe mental illness *n* (%) or mean (SD, *n*)	Diabetes but no severe mental illness *n* (%) or mean (SD, *n*)	Comparison effect size^a^ (C.I.), *p*‐value
Self‐reported health status			
EQ‐5D VAS	56.1 (21.3, 237)	66.1 (21.1, 500)	−8.0 (11.3, −4.6), *p* < 0.001
Quality of life and treatment burden			
Global Quality of Life (WHOQOL‐BREF)	3.1 (1.1, 255)	3.5 (1.1, 500)	−0.3 (−0.5, −0.1), *p* < 0.001
Psychological well‐being (WHO‐5)	38.3 (24.2, 248)	53.9 (24.2, 500)	−13.4 (−17.3, −9.5), *p* < 0.001
Diabetes distress (PAID‐5)	6.4 (5.1, 249)	4.8 (4.5, 500)	1.6 (0.9, 2.3), *p* < 0.001
Impact of diabetes profile (DIDP)			
Composite	3.4 (1.0, 166)	3.9 (1.1, 497)	−0.3 (−0.6, 0.1), *p* < 0.001
Physical health	3.1 (1.2, 249)	3.5 (1.3, 500)	−0.4 (−0.6, −0.2), *p* < 0.001
Financial situation	3.7 (1.3, 235)	3.8 (1.4, 497)	−0.1 (−0.3, 0.1), *p* = 0.39
Leisure activities	3.4 (1.4, 242)	3.6 (1.5, 500)	−0.2 (−0.3, 0.1), *p* = 0.20
Work or studies	3.5 (1.6, 179)	4.6 (2.1, 500)	−0.8 (−1.2, −0.4), *p* < 0.001
Emotional well‐being	3.1 (1.3, 244)	3.6 (1.4, 500)	−0.4 (−0.6, −0.2), *p* < 0.001
Relationship with family, friends and peers	3.8 (1.3, 240)	4.1 (1.4, 500)	−0.3 (−0.5, −0.1) *p* = 0.02
Impact of mental illness profile (DIMP)			
Composite	2.6 (1.1, 170)		
Physical health	2.5 (1.4, 245)		
Financial situation	2.8 (1.5, 237)		
Leisure activities	2.7 (1.4, 237)		
Work or studies	2.7 (1.8, 180)		
Emotional well‐being	2.4 (1.5, 244)		
Relationship with family, friends and peers	3.0 (1.6, 235)		
Empowerment			
DES‐DSF	11.4 (4.4, 251)	12.1 (3.8, 494)	−0.3 (−1.0, 0.3), *p* = 0.30
Self management			
SDSCA‐6 (Average self care days)	3.5 (1.6, 201)	4.5 (1.5, 401)	−0.4 (−0.7, −0.1), *p* = 0.01
Days eat healthy	4.1 (2.6, 250)	5.2 (2.1, 500)	−0.2 (−0.3, −0.1), *p* < 0.001
Days 30 min active	2.6 (2.7, 249)	3.0 (2.5, 500)	−0.01 (−0.1, 0.1), *p* = 0.84
Days testing blood sugar	2.5 (3.2, 249)	3.8 (3.0, 500)	−0.1 (−0.2, −0.03), *p* = 0.01
Days testing blood sugar as recommended	2.8 (3.3, 240)	3.9 (3.0, 500)	−0.1 (−0.2, −0.01), *p* = 0.04
Days checking feet	2.5 (2.8, 248)	3.6 (2.7, 500)	−0.3 (−0.4, −0.2), *p* < 0.001
Days taking medication as agreed	6.4 (1.6, 214)	6.4 (1.4, 401)	0.0 (−0.1, 0.1), *p* = 0.93
Support for self management			
How many people to talk to about diabetes	2.3 (2.7, 243)	3.0 (3.5, 498)	0.7 (0.6, 0.8), *p* < 0.001^b^
Healthcare provision			
PACIC‐DSF	26.0 (11.0, 206)	29.3 (12.4, 495)	−3.3 (−5.4, −1.2), *p* < 0.001
% participating in any diabetes educational programmes or activities	160 (63.4%)	369 (73.8%)	0.7 (0.5, 1.0), *p* = 0.03^c^
Have you smoked a cigarette in the past 7 days?			
Yes, *n* (%)	106 of 250 (42%)	92 of 500 (18%)	2.9 (2.0, 4.3), *p* < 0.001^c^

^a^ Adjusted for diabetes type, age, gender, diabetes duration, disease history and treatment.

^b^ Rate ratio scale.

^c^ Odds ratio scale. See Methods.

### 5.3 Self management behaviours

Although individuals with diabetes and SMI reported similar levels of empowerment as DAWN2‐UK respondents, they were less likely to undertake diabetes self management activities. Specifically, they were less likely to engage in healthy eating, glucose testing and regular foot checks. Smoking rates were 2.3 fold higher in people with SMI. However, there was no difference in activity levels or taking medication as prescribed.

### 5.4 Care and support

People with diabetes and SMI reported receiving less person‐centred care than DAWN2‐UK respondents and were less likely to have an informal carer supporting their diabetes management (57% vs. 66%). A smaller proportion also reported having supportive family relationships (33% vs. 69%), and a greater proportion lived alone (49% vs. 22%).

### 5.5 Diabetes education and information

Fewer individuals with diabetes and SMI reported receiving diabetes education compared with DAWN2‐UK respondents. Among those with diabetes and SMI who had not received such education, the majority (74%) indicated it had never been offered. The disparity was particularly pronounced in relation to structured patient education programmes: 93% of individuals with diabetes and SMI had never participated, compared with 78% of the DAWN2‐UK sample.

## 6 DISCUSSION

The aim of this study was to enhance understanding of the psychosocial impact of comorbid diabetes and SMI. It also sought to examine self management behaviours, care provision, informal support and education. Individuals with diabetes and SMI reported poorer psychosocial outcomes, including lower health‐related quality of life, reduced mental well‐being and elevated diabetes distress, relative to those with diabetes alone. This survey further demonstrated that SMI had a greater negative impact on various domains of daily life than diabetes, which may lead individuals to prioritise their mental health over physical health. This finding aligns with qualitative studies on the lived experience of managing both conditions.^12^ This has implications for motivation to engage in self management, as individuals may be more likely to engage in these behaviours when they perceive diabetes as a priority.^16^ The synthesis of self management patterns, emotional well‐being, perceived support and access to education reveals a complex interplay of factors contributing to health disparities in this group.

Although not all individuals with diabetes engage in all recommended self management behaviours,^13^ people with diabetes and SMI reported even lower engagement levels and were more likely to smoke. While our findings support previous research indicating little difference in medication taking between those with and without SMI,^17^ individuals with SMI reported lower levels of blood glucose monitoring, foot checks and healthy eating, patterns consistent with other surveys of this population.^16^ Prior research has highlighted how the symptoms of SMI, psychotropic medication and their side effects act as barriers to effective self management.^12^ These findings underscore the need to address SMI‐specific challenges to improve diabetes outcomes in this population.

This study also identified disparities in diabetes education and care. Fewer individuals with SMI had received structured, person‐centred education programmes. Importantly, 74% of those who had not undertaken diabetes education reported that it had never been offered, suggesting that lack of access, rather than disengagement, may partly explain this gap. The UK National Diabetes Audit has reported that although 77% of people with type 2 diabetes were offered structured education, only 7.4% attended.^18^ People with SMI may face additional barriers to engagement, including perceiving the information as overwhelming, irrelevant or inaccessible, especially when programmes are not tailored to the needs of individuals with SMI.^19^ These findings indicate that people with SMI may be systematically overlooked in the design and delivery of diabetes education, resulting in an unmet need. Consequently, clinical strategies and education programmes must be adapted to ensure accessibility and relevance for this population.

The study raises an important question regarding where primary responsibility for care should lie. The dual complexity of diabetes and SMI crosses traditional service boundaries, with risks of fragmentation, oversight and unmet need. This underscores the importance of integrated care models, in which mental health services are supported to deliver basic diabetes care, while primary care and diabetes teams are equipped to recognise and manage the additional challenges posed by SMI. Shared responsibility should not rely solely on goodwill or informal coordination, but instead be formalised through collaborative pathways, cross‐training initiatives and co‐located services. Future care should be structured to ensure that individuals with comorbid SMI and diabetes are not excluded by design but are actively included through tailored, person‐centred models that reflect their unique needs.

In terms of informal support, individuals with SMI were more likely to live alone and to report limited support from family, friends and healthcare providers. This reflects broader patterns of social isolation among individuals with SMI, which is associated with poor mental health outcomes, increased morbidity and mortality.^20^ Barriers to involving families in care include patient reservations, staff training needs and the complexity of integrating family support into care plans.^21^ These challenges are likely to affect diabetes care in the same way they impact mental health care.^12^, ^21^ Individuals with SMI also tend to have smaller social networks and, in the absence of a partner or spouse, may rely on professional support services,^22^ which may be inaccessible due to high rates of socioeconomic deprivation.^7^ Nevertheless, family involvement has been identified as a facilitator for diabetes self management and supporting medication‐taking.^23^

Despite higher rates of type 2 diabetes in individuals with SMI, this increased prevalence does not appear to translate into greater access to care or support. Instead, people with SMI experience worse psychosocial outcomes, lower levels of self management and reduced access to diabetes education, highlighting a significant unmet need that current care provision fails to address.

## 7 STRENGTHS AND LIMITATIONS

A major strength of the study is that it examines psychosocial, behavioural and care‐related outcomes together in a population with diabetes and SMI. While previous studies have focused on individual aspects, such as clinical outcomes or mental health burden, this study integrates domains to capture a more holistic view of the lived experience of comorbid diabetes and SMI.

This study utilised the same survey instruments as the DAWN2 study in a cohort of individuals with diabetes and SMI, allowing for direct comparisons with DAWN2‐UK respondents. This enabled a focused examination of the experience and management of diabetes among individuals with SMI. However, the proportion of the eligible population that participated in this study is unknown, limiting our ability to assess the degree of selection bias.

Recruitment challenges in this population may have contributed to self‐selection, potentially resulting in an over‐representation of individuals coping relatively well with their SMI or those accessing healthcare through the mental health or primary care team. We recognised that digital literacy differs among people with SMI, but tried to overcome this by offering the participants multiple ways of completing the survey. It is possible that people who are more engaged in their care may have been more likely to participate, creating a positive selection bias. Conversely, it is also feasible that those with greater difficulties in managing both conditions are overrepresented.

As both samples were cross sectional and the DAWN‐SMI study was undertaken approximately 6 years after the DAWN2 study, causal inferences cannot be drawn and secular changes over time may have influenced the results. There were also differences in the distribution of diabetes types between the two study populations. The DAWN2 study purposively recruited a higher proportion of individuals with type 1 diabetes, resulting in differences in sample composition. Although statistical analyses adjusted for type and potential confounders, it is not possible to rule out the influence of residual or unmeasured factors that may account for some of the observed differences. Furthermore, although using the original DAWN2 questions can be seen as a strength, it is also a possible weakness as newer questionnaires, for example, the 7‐item DAWN2 Impact of Diabetes Profile, may have provided a deeper understanding of the burden of living with diabetes and SMI.

## 8 CONCLUSION

This study confirms that individuals with diabetes and SMI face a higher psychosocial burden than those with diabetes alone, including lower well‐being, reduced quality of life and elevated diabetes distress. The greater impact of SMI on daily life presents distinct challenges to diabetes management, compounded by reduced access to care and education. Whether due to systemic barriers or individual disengagement, the result is a mismatch between need and provision.

Effective diabetes management for people with SMI requires a dual focus on physical and mental health. Care strategies must recognise the interrelated nature of these conditions and be designed with the specific needs of this population in mind. Future research should aim to identify the mechanisms underlying these disparities and inform the development of tailored, accessible interventions to support self management in this underserved group.

## AUTHOR CONTRIBUTIONS

RIGH contributed to the conceptualisation, funding acquisition, investigation, methodology, writing of the original draft and review and editing of the final draft. DB contributed to the methodology, and review and editing of the final draft. HS contributed to the conceptualisation, and review and editing of the final draft. JT contributed to the conceptualisation, data curation, funding acquisition, investigation, methodology, supervision, review and editing of the final draft. NS contributed to the conceptualisation, funding acquisition, investigation, methodology, supervision, and review and editing of the final draft.

## FUNDING INFORMATION

We would like to thank Diabetes UK for funding this study. Project Grant reference: 15/0005447.

## CONFLICT OF INTEREST STATEMENT

RIGH received honoraria for speaker engagement, conference attendance or advisory boards from: Boehringer‐Ingelheim, Eli Lilly, Novo Nordisk, ROVI, USV. He was a member of the international steering committee of the DAWN2 study. HS was a member of the international steering committee of the DAWN2 study.

## ETHICS STATEMENT

This study conforms to the recognised ethical standards and was approved by North East—Tyne & Wear South Research Ethics Committee, REC reference 16/NE/0396.

## Data Availability

The data of the DAWN2 study are property of Novo Nordisk and can be applied for via https://www.novonordisk‐trials.com/en/how‐access‐clinical‐trial‐datasets/. The DAWN2 study is a global partnership initiative for the advancement of person‐centred diabetes care. For more information, contact dawninfo@novonordisk.com. The DAWN‐SMI data collected as primary data for this project are available upon request from the authors and will be deposited and made publicly available in anonymised form latest 12 months after the acceptance of this manuscript.
